# Variations in BK Polyomavirus Immunodominant Large Tumor Antigen-Specific 9mer CD8 T-Cell Epitopes Predict Altered HLA-Presentation and Immune Failure

**DOI:** 10.3390/v12121476

**Published:** 2020-12-21

**Authors:** Karoline Leuzinger, Amandeep Kaur, Maud Wilhelm, Hans H. Hirsch

**Affiliations:** 1 Transplantation & Clinical Virology, Department Biomedicine, University of Basel, Petersplatz 10, CH-4009 Basel, Switzerland; karoline.leuzinger@usb.ch (K.L.); amandeep.kaur@unibas.ch (A.K.); maud.wilhelm@unibas.ch (M.W.); 2 Clinical Virology, Laboratory Medicine, University Hospital Basel, Petersgraben 4, CH-4031 Basel, Switzerland; 3 Infectious Diseases & Hospital Epidemiology, University Hospital Basel, Petersgraben 4, CH-4031 Basel, Switzerland

**Keywords:** BK polyomavirus, large tumor antigen, CD8 T-cell epitope, immune escape, human leukocyte antigen, immunosuppression, transplantation

## Abstract

Failing BK polyomavirus (BKPyV)-specific immune control is underlying onset and duration of BKPyV-replication and disease. We focused on BKPyV-specific CD8 T-cells as key effectors and characterized immunodominant 9mer epitopes in the viral large tumor-antigen (LTag). We investigated the variation of LTag-epitopes and their predicted effects on HLA-class 1 binding and T-cell activation. Available BKPyV sequences in the NCBI-nucleotide (N = 3263), and the NCBI protein database (N = 4189) were extracted (1368 sequences) and analyzed for non-synonymous aa-exchanges in LTag. Variant 9mer-epitopes were assessed for predicted changes in HLA-A and HLA-B-binding compared to immunodominant 9mer reference. We identified 159 non-synonymous aa-exchanges in immunodominant LTag-9mer T-cell epitopes reflecting different BKPyV-genotypes as well as genotype-independent variants altering HLA-A/HLA-B-binding scores. Decreased binding scores for HLA-A/HLA-B were found in 27/159 (17%). This included the immunodominant LPLMRKAYL affecting HLA-B*07:02-, HLA-B*08:01- and HLA-B*51:01-presentation. In two healthy BKPyV-seropositive HLA-B*07:02 blood donors, variant LSLMRKAYL showed reduced CD8 T-cell responses compared to LPLMRKAYL. Thus, despite LTag being highly conserved, aa-exchanges occur in immunodominant CD8 T-cell epitopes of BKPyV-genotypes as well as of genotypes -independent variants, which may contribute to genotype-dependent and genotype-independent failure of cellular immune control over BKPyV-replication. The data warrant epidemiological and immunological investigations in carefully designed clinical studies.

## 1. Introduction

BK polyomavirus (BKPyV) is an opportunistic pathogen causing polyomavirus-associated hemorrhagic cystitis (PyVHC) in 5–20% of allogeneic hematopoietic stem cell (HSCT) recipients [1,2,3] and polyomavirus-associated nephropathy (PyVAN) in 1–15% of kidney transplant (KT) patients [4,5]. Moreover, PyV-associated urothelial cancer (PyVUC) has been recognized as an emerging entity in immunosuppressed patients with a history of prolonged BKPyV-replication and nephropathy following accidental integration of the BKPyV genome into human host cell chromosomes [6,7,8,9]. Uncontrolled BKPyV replication due to insufficient BKPyV-specific immunity appears to be the common denominator of these three major BKPyV diseases [10,11,12,13]. Conversely, immunocompetent individuals remain without significant illness despite high BKPyV infection rates starting in early childhood and reaching seroprevalence rates of >90% in the general adult population [14,15]. After primary infection, BKPyV persists in the renourinary tract and, despite potent BKPyV-specific T-cells and neutralizing antibodies (NAbs) [13], immunocompetent healthy blood donors show low-level urinary BKPyV shedding indicating effective escape from immune control [16]. BKPyV immune escape is favored by the viral agnoprotein which actively interferes with innate immune sensing, hence preventing the timely alarming of the adaptive immune response [17]. Following transplantation, BKPyV-replication increases in rates and magnitude in the KT-recipients as a result of immunosuppression, HLA-mismatches, and preferentially involves donor-derived BKPyV genotypes [18,19,20]. In addition, low or absent NAb titers against the donor BKPyV-genotype have been associated with an increased risk of developing BKPyV-DNAemia and nephropathy after kidney transplantation [21,22,23]. Similar to rearrangements of the viral non-coding control region [24], amino acid (aa) exchanges in the BKPyV-Vp1 capsid, and specifically in its BC-loop, have been observed in patients with prolonged BKPyV replication periods. Some variability in the viral DNA-genome may arise by deamination of the antisense strand by the apolipoprotein B editing complex (APOBEC) 3, thereby introducing point mutations in the *VP1*-gene encoding the major viral capsid protein Vp1 [25]. Although antibody titers to BKPyV virus-like particles generally correlate with BKPyV-specific CD4 T-cells [26], some reports indicate lack of correlation of plasma BKPyV loads with the emergence of BC-loop mutations or with rising NAb titers [27,28]. Although immune control at the level of the viral capsid, and specifically by BKPyV-Nabs, is likely to be important in protecting from systemic spread of the virus, recent data have demonstrated that plasma BKPyV loads in KT patients do not result from BKPyV virions [29], but mostly represent DNAse-sensitive, unprotected genome fragments similar to what has been reported for cytomegalovirus (CMV) [30,31]. Unlike described for enveloped viruses carrying viral membrane proteins, BKPyV-capsid antibodies cannot eliminate infected host cells replicating the non-enveloped virions by antibody-dependent cytotoxicity, but rather interfere after virus release with new rounds of infection. Moreover, access of NAbs to renal tubules and their effective blocking of viral cell-to-cell spread inside the renal tubules is presently unresolved [13]. In view of the strong antiviral T-cell responses [32,33], we and others have focused on characterizing BKPyV-specific CD8 T-cells [34,35,36]. We found that clearance of BKPyV-DNAemia is associated with increasing BKPyV-specific CD8 T-cell cytotoxic responses to immunodominant 9mers [37]. Notably, the 9mers clustered in hot spots of the viral large T-antigen (LTag) [37], some of which could be independently linked to human leukocyte antigen (HLA)-types such as HLA-B7, HLA-B8 and HLA-B51, partially protecting from BKPyV-DNAemia [38,39]. Since BKPyV-variants can emerge in KT patients with ongoing viral replication [23,24], we hypothesize that non-synonymous aa-exchanges in immunodominant LTag T-cell epitopes may contribute to failing immune control over BKPyV replication. We therefore addressed the question of whether or not aa-exchanges can occur in the BKPyV LTag, and if so, whether or not such variants affect previously characterized immunodominant 9mer-epitopes of relevance for cytotoxic T-cell control, adoptive T-cell transfer, and vaccine development [40].

## 2. Materials and Methods

### 2.1. Assessing Variation in the BKPyV LTag Sequence

The NCBI nucleotide and protein database is a collection of sequences from several sources, including nucleotide sequences and translations from annotated coding regions in GenBank and RefSeq as well as records from SwissProt, the Protein Information Resource (PIR), and Brook-haven Protein Data Bank (PDB). Sequences were aligned against the *LTAG*-gene and LTag-protein sequence of the BKPyV-WW reference genome (BKPyV subtype Ib-1; acc. no.AB211371.1), yielding 521 complete and 121 partial BKPyV *LTAG*-nucleotide sequences from the NCBI nucleotide database, and 314 complete and 412 partial BKPyV LTag-protein sequences from the NCBI protein database. The 521 complete BKPyV *LTAG* sequences were processed using “reverse complement sequence”, as they were derived from full-length BKPyV genome sequences and had anti-sense orientation. Nucleotide sequences were translated into protein sequences using the “translate to protein” tool. All bioinformatic analysis was done using the CLC Genomic Workbench software (version 12; QIAGEN, Hilden, Germany).

### 2.2. BKPyV Genotypes, BKPyV-Variants and LTag-9mer Variants

BKPyV can be categorized into four major genotypes (BKPyV I, II, III and IV) based on neutralization and the specific sequences in the major BKPyV capsid gene *VP1* [41], but current genotyping relies on *VP1*- and *LTAG*-sequences to identify subtypes Ia, Ib1, Ib2, Ic, II, III, and IVa1, IVa2, IVb1, IVb2, IVc1, and IVc2 [5,23,42,43]. BKPyV-variants were defined as changes in any aa residue not attributable to a BKPyV genotype. LTag-9mer variants were defined as aa-exchanges in previously reported immunodominant 9mer T-cell epitopes [34,37].

### 2.3. Prediction of HLA-A and -B Binding of BKPyV Immunodominant LTag 9mer T-Cell Epitopes

The Immune Epitope Database and Analysis Resource tool (IEBD) was used to predict the binding score of wildtype and variant immunodominant 9mer LTag T-cell epitopes [34,37] for the 14 most prevalent HLAs in Europe and North America. To study the impact on HLA-binding of these variants, we focused on HLA-B7, -B8, -51, which have been described to present immunodominant LPLMRKAYL epitope [38] using HLA-A24 as control not binding this epitope. A threshold difference of 0.05 in the HLA-binding score was interpreted as significant change in binding.

### 2.4. BKPyV-Specific CD8 T-Cell Responses In Vitro

CD14+ cells were isolated from PBMCs of two healthy BKPyV IgG-seropositive blood donors (donor 1 HLA-A26/28, HLA-B07/55 and donor 2 HLA-A03/03, HLA-B07/07) and differentiated into mature monocyte-derived dendritic cells (mMo-DCs) as described [40]. mMo-DCs were pulsed with LTag overlapping 27mer pool and co-cultured with autologous CD14- cells for 9 days. After expansion, cells were re-stimulated with wildtype 9mer peptides (9mP), a pool of 97 immunodominant wildtype or variant 9mP, in which solely the wildtype 9mer127 (LPLMRKAYL) was replaced by variant 9mer127 (LSLMRKAYL); with wildtype 9m127 or with variant 9mer127 alone, or with wildtype 9mer126 (NLPLMRKAY) or variant 9mer126 (NLSLMRKAY).

### 2.5. Statistical Analysis

All statistical data analysis was done in R (v3.6.1; https://cran.r-project.org), and Prism (v8; Graphpad Software, San Diego, CA, USA) was used for data visualization. Statistical comparison of non-parametric data was done using Mann–Whitney U test.

## 3. Results

### 3.1. Identification of BKPyV LTag Variants

**Figure 1:**
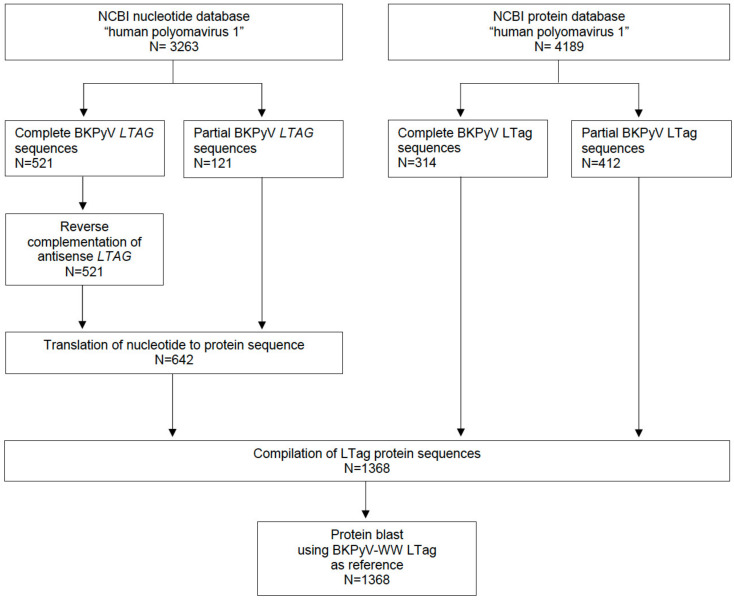
Bioinformatic analysis flowchart. BKPyV large tumor antigen (LTag) sequences were downloaded from the NCBI nucleotide and protein database (as of 13 November 2020). Nucleotide Scheme 12. QIAGEN, Hilden, Germany) and the LTag BKPyV-WW sequence as reference (BKPyV subtype Ib-1; acc. no.AB211371.1).

Sequences of BKPyV, also known as human polyomavirus 1, were retrieved from the NCBI nucleotide database (N = 3′263; as of 13 November 2020), and the NCBI protein database (N = 4′189; as of 13 November 2020; Figure 1). A total of 1368 protein sequences were compiled, and protein blast was done using the LTag-protein sequence of BKPyV-WW as reference (acc. no.AB211371.1).

**Figure 2:**
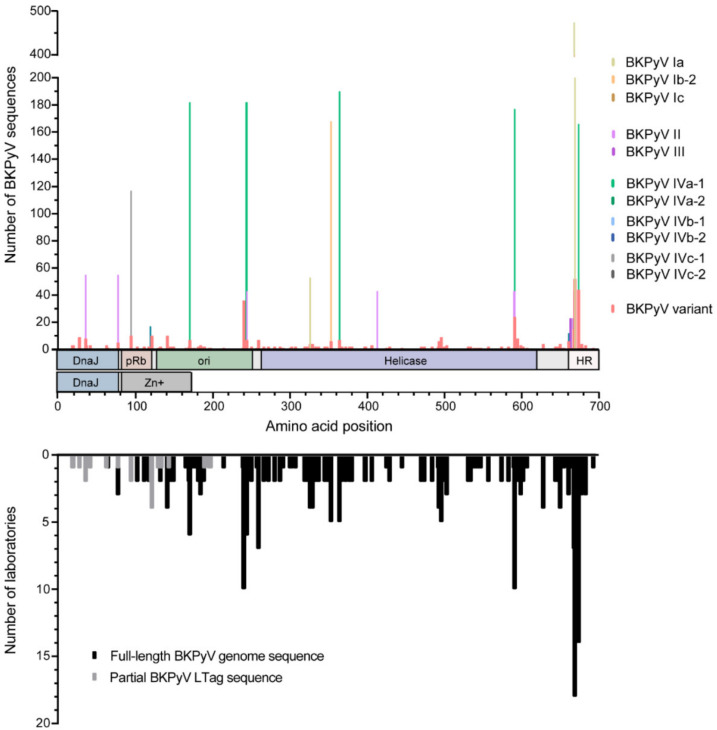
Variation in the BKPyV LTag protein sequence. BKPyV subtype specific variation and amino acid exchanges not related to a certain BKPyV subtype (i.e., BKPyV variants) in the large tumor antigen (LTag) protein sequence were identified (upper panel); number of laboratories that deposited full-length BKPyV genome or partial LTag sequences from BKPyV-variants (lower panel). Schematic drawing of the respective domains in LTag and small T antigen (sTag; according to DeCaprio and Garcea [44]) are indicated below the diagram (DnaJ homology region (DnaJ), retinoblastoma protein binding domain (pRb), origin of DNA replication binding domain (ori), host range domain (HR)). Of note, the amino-terminal part including the DnaJ homology domain is identical for sTag and LTag, whereas the carboxyterminal domains indicated are specific for sTag or LTag.

Although the LTag-protein sequence is highly conserved among the different BKPyV subtypes, genotype-associated aa were identified, and were therefore called genotype aa-signature positions (Figure S1, **Table S1**). These included in BKPyV subtype *Ia*: T327I; in BKPyV subtype *Ib-2*: L670S; T354S, L670S; in BKPyV subtype *Ic*: L670S; in BKPyV subtype *II/III*: R36K, S78N, T245I, T327I, A591S, T592K, D671N, and Q668E in BKPyV subtype *III* only; in BKPyV subtype *IV*: S95R, A120G, Q171L, H244Y, T245I, E365D, I414V, T592Q, A662G, L670V and Q675E. In addition, aa-exchanges were identified defining LTag-variants not associated with a specific BKPyV-genotype (Figure 2). Such genotype-independent variants were located in the DnaJ-homology region (DnaJ) shared between LTag and sTag, in the retinoblastoma protein binding domain (pRb), the origin of DNA replication binding domain (ori) and the helicase domain. Moreover, some aa-exchanges clustered with >10 BKPyV sequences for example in the C-terminus of the ori- domain, in the C-terminus of the helicase- and in the host range-domain. Notably, many variant BKPyV-LTag sequences were independently identified in full-length BKPyV genomes submitted by five to 18 different laboratories (Figure 2).

### 3.2. Identification of Amino Acid Exchanges in Immunodominant LTag 9mer T-Cell Epitopes

**Figure 3:**
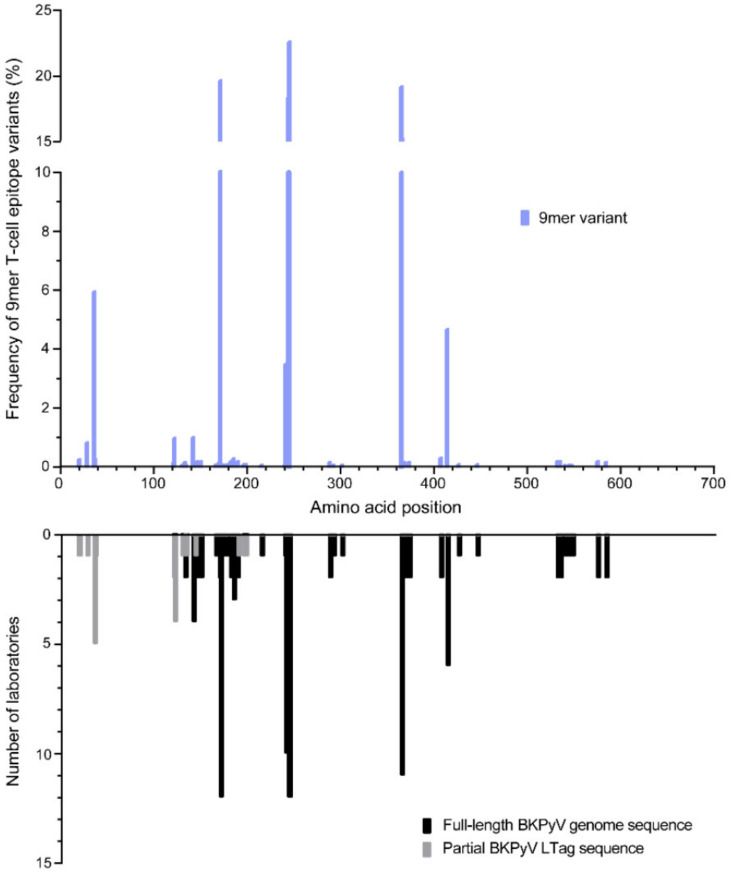
Frequency of aa-exchanges in 97 immunodominant LTag-9mer T-cell epitopes. Amino acid exchanges in 97 previously reported immunodominant large tumor antigen (LTag) 9mer T-cell epitopes [34,37] were identified (upper panel) and number of laboratories (lower panel) that reported the respective change in full-length BKPyV genome (black) or in partial LTag sequences (grey).

Given the role of the 97 immunodominant LTag-9mer T-cell epitopes described previously [34,37], we investigated whether or not immunodominant epitopes were altered by the aa-exchanges identified. Indeed, 159 aa-exchanges occurred in the LTag-9mer epitopes with differing frequencies (Figure 3 and Table 1). Of these, 127 LTag-9mer variants occurred with frequencies <1%, 14 with frequencies of 1% to <5%, 10 with frequencies of 5% to <10% and 8 with frequencies of 10% to <25% (Table 1). The most frequent LTag-9mer variants were identified in the N-terminus of the DnaJ domain with frequencies up to 6%, in the N- and C-terminus of the ori domain with frequencies up to 23%, and in the helicase domain with frequencies up to 19% (Figure 3). These 9mer variants had been independently submitted as partial and full-length BKPyV genome sequences from 6 to 12 different laboratories. Remarkably, the highest frequencies of the 9mer variants above 18% were noted at LTag-positions 171, 244, 245 and 365 and independently reported from more than ten different laboratories, and reflected BKPyV genotype IV. LTag-9mer variants at position 36 and 414 with frequencies of around 5%, were associated with BKPyV genotype II and III. LTag-9mer variants not associated with a certain BKPyV subtype had been identified with the highest frequency of 4% at position 241, while most other LTag-9mer variants displayed frequencies <2%. Together, the data indicated that the highly conserved LTag carried aa-exchanges in immunodominant 9mer T-cell epitopes that were BKPyV-genotype specific or were genotype-independently diversified through aa-exchanges.

### 3.3. LTag-9mer Variants Affect HLA-B*07:02, HLA-B*08:01 and HLA-B*51:01 Binding

**Figure 4:**
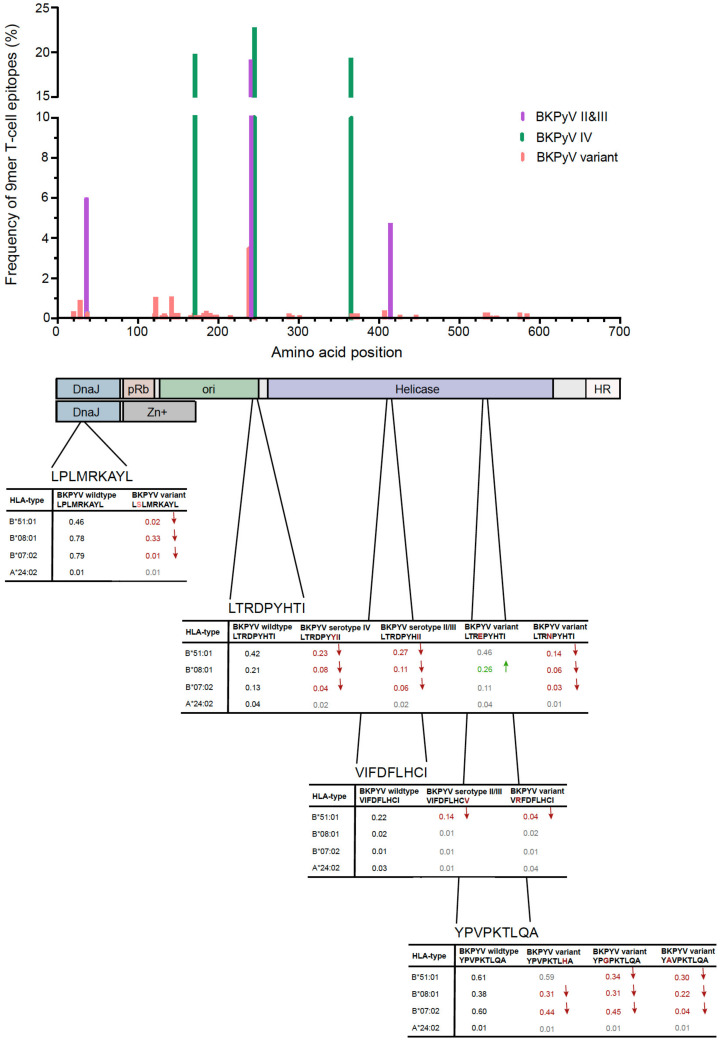
BKPyV-genotype independent amino acid exchanges in immunodominant LTag-9mer T-cell epitopes on HLA-A and HLA-B binding. The indicated BKPyV variant sequences occurred in previously reported LTag-9mer T-cell epitopes [34,37]. HLA binding was predicted using the Immune Epitope Database and Analysis Resource tool (red ↓ indicates a significant decrease in binding of at least 0.05; green ↑ indicates a significant increase in binding of at least 0.05). Schematic drawing of the respective domains in LTag and small T antigen (sTag; according to DeCaprio and Garcea [44]) are indicated below the diagram (DnaJ homology region (DnaJ), retinoblastoma protein binding domain (pRb), origin of DNA replication binding domain (ori), host range domain (HR)). Of note, the amino-terminal part including the DnaJ homology domain is identical for sTag and LTag, whereas the carboxyterminal domains indicated are specific for sTag or LTag.

Genotype-independent aa-exchanges occurred at 55 individual LTag positions in immunodominant 9mer T-cell epitopes, and thus, occurred more frequently than aa related to BKPyV genotype II, III and IV found at six LTag positions (*p* < 0.001; Figure 4). However, BKPyV genotype specific aa-exchanges occurred with higher frequencies than aa-exchanges related to BKPyV variants (median frequency of 18.2% (±6.1% 95%CI) vs. 0.2% (±0.4% 95%CI); *p* < 0.001; Figure 4).

Next, we investigated whether or not such changes were predicted to be associated with decreased HLA-presentation and possibly impaired T-cell control. To this end, we applied the Immune Epitope Database and Analysis Resource tool analyzing changes in the binding score of the LTag-9mers carrying aa-exchanges linked to a specific BKPyV-serotype or BKPyV-serotype independent variants. Several aa-exchanges were associated with a significant change of the binding score to HLA-A and HLA-B (Figure S2). Overall, 27/159 (17%) LTag-9mer variants showed a significantly decreased binding score, while 18/159 (11%) LTag-9mer variants increased binding scores. For 3/159 (2%) LTag-9mer variants, both increased and decreased bindings scores were predicted affecting different HLA-types (Table 1).

Specifically, aa-exchange I414V (frequency of 4.7%) was associated with BKPyV subtype II/III, and led to decreased HLA-A/HLA-B binding of the BKPyV-serotype I-encoded peptide VIFDFLHCV, but did not have an effect on the HLA-binding of other LTag-9mer variants such as FDFLHCVVF, FLHCVVFNV and VVFNVPKRR (Figure S2 and Figure 4). Similarly, the P534A exchange (0.1%; BKPyV variant) affected the MHC-anchor position of the LTag 9mer epitope 9m533 YPVPKTLQA [37] with starting position 533 and dramatically decreased the binding score for HLA-B*07:02, HLA-B*08:01 and HLA-B*51:01. When the aa-exchanges occurred at positions other than P534A, the binding score was also decreased for HLA-B*07:02 and HLA-B*08:01 compared to the reference YPVPKTLQA LTag-9mer T-cell epitope, but the effects were less pronounced compared to the specific 9mer anchor position (Figure S2 and Figure 4).

To analyze in detail the aa-exchanges at specific anchor positions of MHC-class I, we focused on the previously well characterized immunodominant 9m127 T-cell epitope LPLMRKAYL having its starting position at LTag aa position 27 (Figure 4). The LTag-9mer variant 9m127 LSLMRKAYL is not associated with a specific BKPyV-genotype, and occurred with a low frequency of 0.9%, being independently reported from 2 different laboratories (Figure 3). Interestingly, the P28S aa-exchange did not change the predicted low HLA-presentation of the overlapping LTag-9m126 NLSLMRKAY or -9m128 SLMRKAYLR [37] (Figure S2).

Based on the clinical study by Leboeuf et al. [37], relevant examples of BKPyV-genotype independent variants causing aa-exchanges that impair HLA-class I-specific anchor positions and corresponding predictions of immunodominant 9mer T-cell epitopes were found at LTag-starting positions 406 and 533 (Figure 4 and Figure S2). Interestingly, some aa-exchanges occurring in other than anchor positions were also significantly impacting the binding score of wildtype 9mers, as in the LTag 9m328 LTRDPYHTI. The variants LTRDPYYII and LTRDPYHII were predicted to decrease HLA-B*07:02, B*08:01, and most dramatically HLA-B*51:01 binding impairing T-cell receptor binding and activation. In contrast, aa-exchanges at position 241 (LTREPYHTI) had little effect on HLA-binding, and even appeared to slightly increase the binding score for HLA-B*08:01 compared to BKPyV LTag-9mer reference (Figure 4).

**Figure 5:**
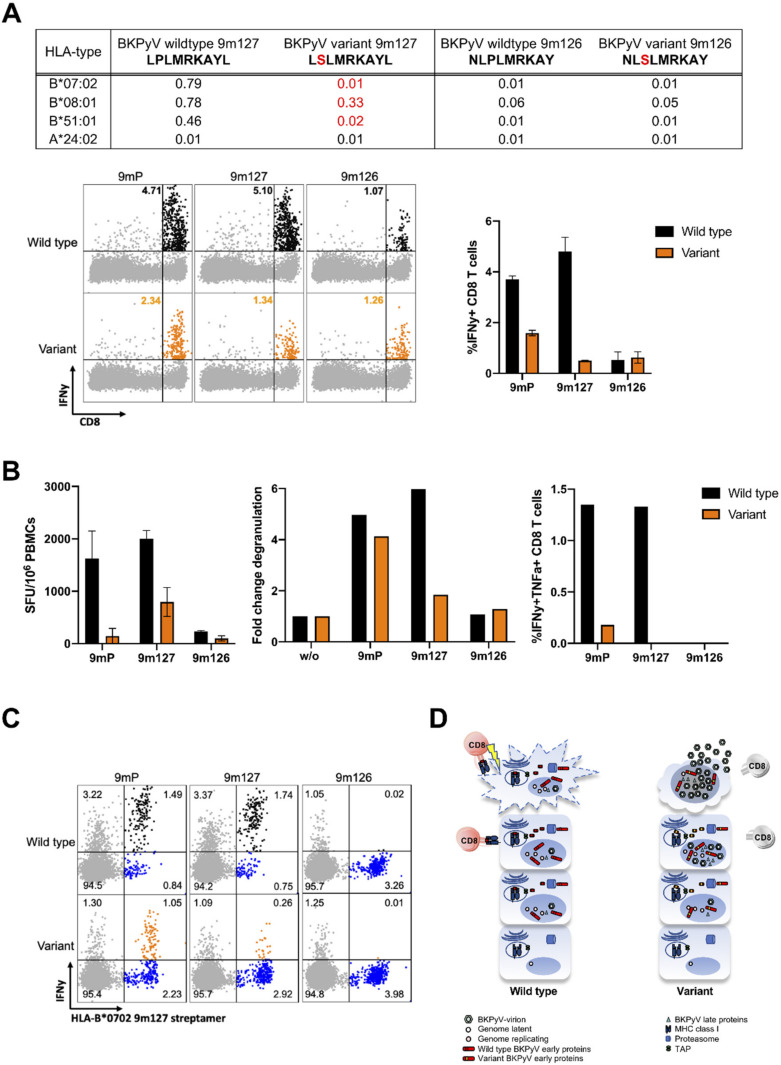
Comparing CD8 T-cell responses to wildtype and variant 9mer127. (**A**) Top: Predicted HLA-binding scores for wildtype 9mer127 and 9mer126 as well as their respective variants. Bottom: Representative flow-cytometry and intracellular cytokine staining (ic-FACS) for interferon-(IFN)γ (left) and cumulative results of two healthy BKPyV-IgG-seropositive HLA-B07-positive blood donors (right) showing the percentage of IFNγ + CD8 T-cells after re-challenge with wildtype (black) or variants peptides (orange). (**B**) BKPyV-CD8 T-cell function was independently evaluated for IFNγ by 9mer-ELISpot (left); or for CD107a+ IFNγ+ CD8 T-cell degranulation responses (middle); or by ic-FACS for polyfunctional TNFα+ IFNγ+ CD8 T-cell responses (right). (**C**) HLA-streptamer staining of CD8 T-cells was performed using PE-labelled streptactin with HLA-B*0702 molecules bearing wildtype 9m127 peptide as described [37,40], comparing response to wildtype or variant peptides. (**D**) Model of immune control or failure of BKPyV-specific CD8 T-cells to wildtype or variant aa-exchanges in immunodominant LTag-specific 9mer epitopes. The early viral gene region-encoded LTag is shown in red, late viral gene region-encoded proteins such as the viral capsid proteins are shown in green. The host cell proteasome processes viral proteins generating small peptides which are transported into the endoplasmic reticulum (ER) via the transporter-associated with antigen processing (TAP). Left: In case of BKPyV encoding the wildtype LTag, immunodominant epitopes are able to stably bind to the respective MHC class I molecule in the ER. The MHC class I- epitope complex is transported via the secretory apparatus to the cell surface. BKPyV-specific CD8 T-cells recognizing the MHC class I-epitope complex via its T-cell receptor and CD8. Activated BKPyV-specific CD8 T-cells kill the BKPyV-replicating host cells at an early stage of viral replication. Right: In case of BKPyV encoding variant LTag, the variant epitopes cannot properly bind to MHC class I in the ER. As a result, this epitope MHC complex is unstable and not transported to the cell surface and recognition and killing by BKPyV-specific CD8 T-cells cannot occur, permitting uncontrolled BKPyV-replication.

Given the implications of these predictions on CD8 T-cell function, we conducted a functional study comparing CD8 T-cell responses to the immunodominant 9mer127 LPLMRKAYL and the corresponding variant LSLMRKAYL in PBMCs from two healthy BKPyV IgG-seropositive blood donors carrying HLA-B*07:02 allele using an established protocol [40]. Using flow-cytometry and intracellular cytokine staining (ic-FACS) for interferon-(IFN)γ, the responses to the variant 9mer127 were significantly reduced, whereas the responses to 9m126 wildtype NLPLMRKAY or variant NLSLMRKAY remained similar (Figure 5A). When replacing the wildtype 9m127 with the variant in the 9mer pool (9mP) consisting of 97 immunodominant 9mers [37,40], the IFNγ-responses were also reduced but well detectable as expected (Figure 5A). More detailed characterization of donor 1 revealed reduced IFNγ-responses in the 9mer-EliSpot as an independent assay (Figure 5B, left panel); and reduced polyfunctional responses to the variant 9m127 by ic-FACS for IFNγ, tumor-necrosis-factor-(TNF)α and CD107a-degranulation (Figure 5B, middle and right panel). To examine the contribution of HLA-B*07:02-specific CD8 T-cells to IFNγ-responses, the corresponding streptamers were used as described [37,40], after stimulation with wildtype or variant 9mer127 peptides (Figure 5C, left panel). The results revealed reduced responses to the variant 9mP, and low or no responses to the variant 9mer127 or 9mer126. Taken together, the results provided first functional evidence for a model of reduced CD8 T-cell control to variants or immunodominant 9mer-epitopes (Figure 5D) implicated in cellular BKPyV-immune control [38,39].

## 4. Discussion

BKPyV-specific T-cells play a key role in antiviral immune control [13,32,45,46]. Recent work from our group pinpointed clusters of immunodominant 9mer epitopes in the viral LTag as relevant targets of BKPyV-specific T-cells [34,37,40]. In the present study, we investigated aa-exchanges in LTag, and predicted effects of the resulting 9mer epitopes on HLA-A and HLA-B binding scores. Our study has three major findings:

First, BKPyV genotype-specific aa-signatures can be identified in the otherwise structurally and functionally highly conserved LTag. The frequency of these aa-signatures corresponds to those reported for BKPyV-subtypes in numerous studies with BKPyV I being most frequent, followed by type IV, and less frequently II and III [16,23,43,47,48]. Although BKPyV-LTag is highly conserved among the BKPyV genotypes and related polyomaviruses, the genotype-specific aa represent sequence signatures allowing for specific attribution to the respective LTag-protein and imply that the encoding genotype virus is functional and transmitted.

Second, certain genotype-specific aa-signatures affect immunodominant 9mer peptides presented by HLA-A/HLA-B to BKPyV-specific T-cells. Thereby, a decrease in the HLA-binding score of the corresponding LTag-9mer epitope is predicted compared to the BKPyV-genotype I reference. Examples are the LTag-9mer epitopes VIFDFLHCV and LTRDPYHII associated with BKPyV-genotype II and III or LTag-9mer epitope LTRDPYYII encoded BKPyV-genotype IV. The data suggest the testable hypothesis that first exposure to a BKPyV-subtype is associated with an HLA- and epitope-specific T-cell response typically associated with cellular immune control in the general healthy population. However, secondary exposure to a different subtype through kidney transplantation may be associated with an impaired T-cell control in addition to immunosuppression. Similarly, novel increases in HLA-binding scores of new subtype or variant 9mer-epitopes may not be met by matching CD8 T-cell memory or actually being 9mer-epitope naïve. A similar failure scenario can be envisaged for adoptive T-cell transfer or for allogeneic HSCT. Our observations would potentially also account for hitherto little understood clinical cases having high persisting plasma BKPyV loads despite detectable BKPyV-specific T-cell responses in assays based solely on subtype-I peptides.

Third, aa-exchanges were identified in positions not attributable to a specific BKPyV-genotype, but affected the binding score of genotype-independent immunodominant LTag-9mer epitopes. In particular, critical aa-exchanges at MHC class I-specific anchor positions of immunodominant LTag T-cell epitopes (e.g., LPLMRKAYL to LSLMRKAYL) significantly decreased the binding score of HLA-B*07:02, HLA-B*08:01, HLA-B*51:01. Our data from two healthy BKPyV IgG-seropositive blood donors support the predicted effects of failing CD8 T-cell function when changing the 9mer peptide from LPLMRKAYL to LSLMRKAYL in assays such as 9mer-EliSpot and ic-FACS for IFNγ, tumor-necrosis-factor-(TNF)α and CD107a-degranulation (Figure 5). The data suggest that BKPyV-variants in the LTag may account for genotype-independent failure of cellular immune control, whereby such variants may emerge during on-going viral replication escaping immune selection pressure (Figure 5D).

Although our data call for dedicated clinical studies independently evaluating the epidemiology, the risk factors, and the functional consequences of LTag aa-exchanges identified here, we note that all relevant aa-exchanges have been reported by several laboratories, hence rendering outliers and sequencing artefacts less likely. Most aa-exchanges were located close to pre-defined functional LTag domains. While we currently cannot yet predict effects on BKPyV replication, their presence in clinical samples argues for validity and in favor of clinical and biological significance. A large proportion of the BKPyV data were obtained from full-length genome sequences implying a dedicated setting which, together with its submission to public databases, may suggest high data quality. Moreover, the full-length BKPyV genome sequences enabled us to compare relative frequencies across different positions, unlike for partial sequences mostly submitted for diagnostic, clinical or subtyping reasons. The majority of BKPyV sequences available in the NCBI nucleotide and protein database were derived from BKPyV genotype I and IV. This distribution pattern is consistent with serological studies indicating that BKPyV genotype I is found in 60–70% of patients worldwide, followed by genotype IV in 10–20% of blood donors and transplant patients alike [43,47,48], while genotypes II and III are generally rare [16,23].

While significant immunological research focuses on the Vp1-capsid protein of BKPyV and related HPyVs, we observed in kidney transplant patients clearing BKPyV DNAemia that the proportion of BKPyV-specific CD8 T-cells is greater among LTag-specific cellular immune responses than the one among Vp1-specific cellular responses [11]. The relevance of CD8 T-cells directed to LTag was independently confirmed by others as reviewed [13] and supported in our follow-up study [37]. From the virological perspective, incepting viral replication at an early time point may be advantageous in viral progeny control as compared to late stages, when high numbers of virions have readily accumulated and possibly leak from the host cell nucleus [13]. Our recent work suggests that alarming innate and adaptive immune responses may be delayed in the late phase of BKPyV replication [17]. Finally, we speculate that LTag-targeted CD8 T-cell responses including those directed against the N-terminus shared with small T-antigen [37] may protect against BKPyV-associated urothelial carcinoma expressing solely LTag or truncated derivatives from chromosomally integrated BKPyV genomes [8,9], when there is little or no Vp1 expression [7].

Taken together, our results add another layer to interpreting the increased risk of BKPyV-DNAemia and nephropathy in KT patients in donor–recipient pairs having BKPyV-serotype mismatch [5], which has been solely attributed to lacking the corresponding genotype-specific NAbs [18,21]. Conversely, high NAbs in the recipient against the donor genotype may be a marker for matching genotype-specific T-cells [26], adding to the partial protection and earlier clearance of plasma BKPyV loads in many [22,49,50], but not all cases [28]. Other observational studies can also be discussed in the new light of our findings, which report significant correlations of decreasing BKPyV-specific T-cells from pre-transplant to post-transplant with increased risk [12]. Increasing BKPyV-specific T-cells were associated with shorter duration, decline, and clearance of BKPyV-viremia [11,33,51,52]. Re-analyzing the role of LTag-subtypes and -variants will be of interest in this context.

## 5. Conclusions

Despite a high degree of conservation of LTag, aa-exchanges occur in LTag-9mer CD8 T-cell epitopes that significantly alter the predicted HLA-A/HLA-B-presentation of BKPyV- genotypes and -variants and potentially impact BKPyV-specific T-cell activation and function. Genotyping BKPyV, NAbs, and emerging variants in transplant patients may provide novel direct and indirect information about BKPyV-specific LTag CD8 T-cell responses as markers of immune control. The data warrant further epidemiological and immunological investigations in carefully designed clinical studies.

## Figures and Tables

**Table 1:** Altered HLA-A and HLA-B binding of wildtype and variant LTag-9mer T-cells epitopes.

Frequency of LTag 9mer Variants (%)	Number of LTag 9mer Variants	Number of LTag 9mer Variants with Changed HLA Binding ^1^			
		↓	↑	↓↑	Total
<1%	127	19	15	2	36
1–<5%	14	4	1	1	6
5–<10%	10	2	1	-	3
10–<25.0%	8	2	1	-	3

^1^ To address the impact of reported aa exchanges on HLA-presentation, we applied the Immune Epitope Database and Analysis Resource tool analyzing changes in the binding score of HLA-B*07:02, HLA-B*08:01, HLA-B*51:01 and HLA-A*24:02. A threshold difference of 0.05 between the wildtype and variant LTag 9mer T-cell epitope HLA-binding score was interpreted as significant change in binding (↓ indicates a significant decrease in binding of at least 0.05; ↑ indicates a significant increase in binding of at least 0.05).

## Supplementary Materials

The following are available online at https://www.mdpi.com/1999-4915/12/12/1476/s1, Figure S1. LTag sequence conservation among BKPyV subtypes; Figure S2. BKPyV LTag-9mer T-cell immune escape map. Table S1. Sequence conservation of BKPyV LTag.
