# Supplementary material for: Variations in BK Polyomavirus Immunodominant Large Tumor Antigen-Specific 9mer CD8 T-Cell Epitopes Predict Altered HLA-Presentation and Immune Failure
Source: Viruses. 2020 Dec 21;12(12):1476. doi: 10.3390/v12121476 (PMC7767524; doi:10.3390/v12121476)
Supplement: Supplementary file 1 [file viruses-12-01476-s001.zip › viruses-1034944 supplementary update/caption.pdf]

Figure S1. LTag sequence conservation among BKPyV subtypes;

Figure S2. BKPyV LTag-9mer T-cell immune escape map. Table S1. Sequence conservation of BKPyV LTag.
