# Supplementary material for: Variations in BK Polyomavirus Immunodominant Large Tumor Antigen-Specific 9mer CD8 T-Cell Epitopes Predict Altered HLA-Presentation and Immune Failure
Source: Viruses. 2020 Dec 21;12(12):1476. doi: 10.3390/v12121476 (PMC7767524; doi:10.3390/v12121476)
Supplement: Supplementary file 1 [file viruses-12-01476-s001.zip › viruses-1034944 supplementary update/Figure_S2.pdf]

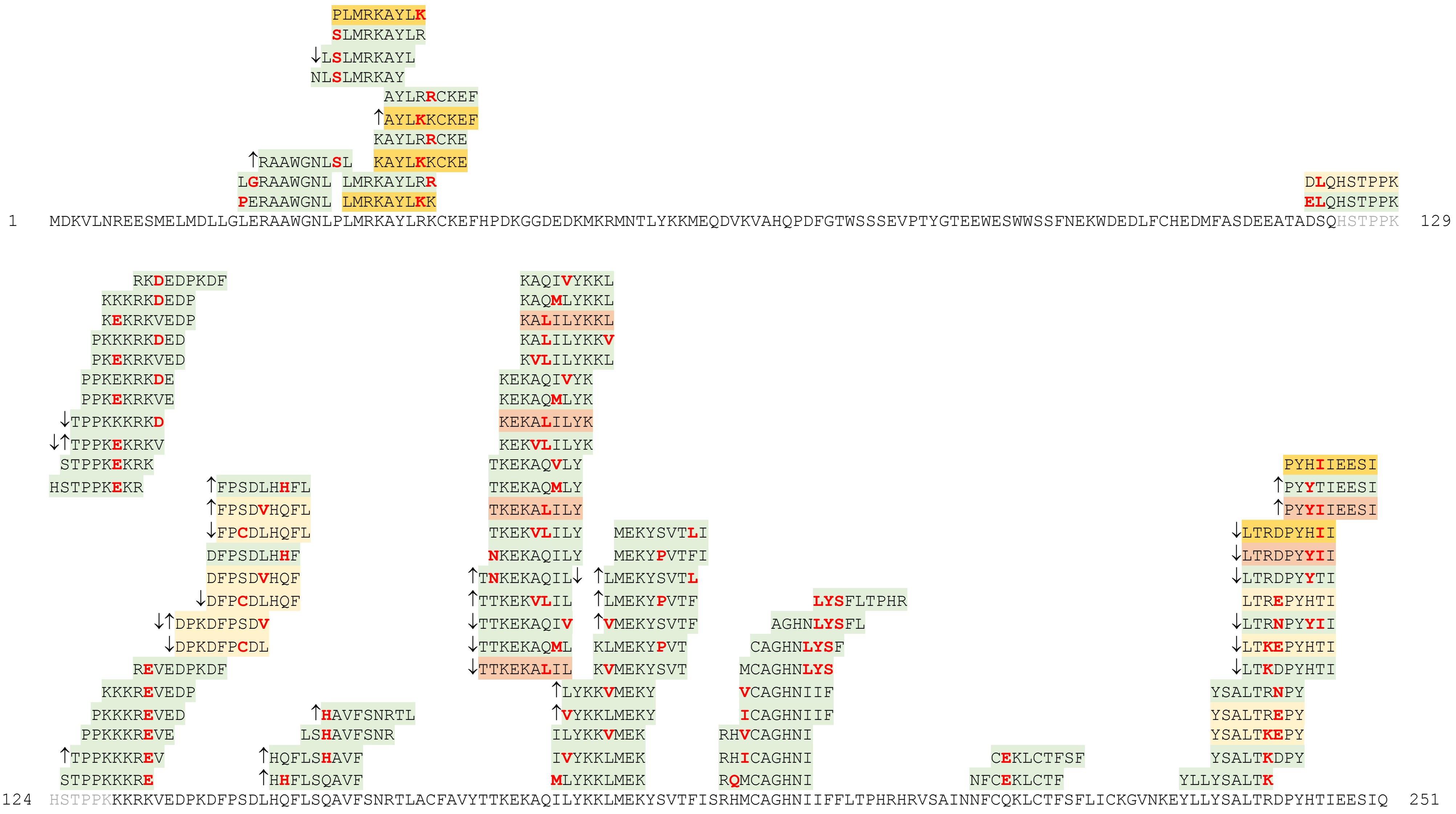
PLMRKAYLK
SLMRKAYLR
↓LSLMRKAYL
NLSLMRKAY
AYLRRCKEF
↑AYLKKCKEF
KAYLRRCKE
↑RAAWGNLSL KAYLKKCKE
LGRAAWGNL LMRKAYLRR DLQHSTPPK
PERAAWGNL LMRKAYLKK ELQHSTPPK
1 MDKVLNREESMELMDLLGLERAAWGNLPLMRKAYLRKCKEFHPDKGGDEDKMKRMNTLYKKMEQDVKVAHQPDFGTWSSSEVPTYGTEEWESWWSSFNEKWDEDLFCHEDMFASDEEATADSQHSTPPK 129
RKDEDPKDF KAQIVYKKL
KKKRKDEDP KAQMLYKKL
KEKRKVEDP KALILYKKL
PKKKRKDED KALILYKKV
PKEKRKVED KVLILYKKL
PPKEKRKDE KEKAQIVYK
PPKEKRKVE KEKAQMLYK
↓TPPKKKRKD KEKALILYK
↓↑TPPKEKRKV KEKVLILYK
STPPKEKRK TKEKAQVLY PYHIIEESI
HSTPPKEKR ↑FPSDLHHFL TKEKAQMLY ↑PYYTIEESI
↑FPSDVHQFL TKEKALILY ↑PYYIIEESI
↓FPCDLHQFL TKEKVLILY MEKYSVTLI ↓LTRDPYHII
DFPSDLHHF NKEKAQILY MEKYPVTFI ↓LTRDPYYII
DFPSDVHQF ↑TNKEKAQIL↓ ↑LMEKYSVTL ↓LTRDPYYTI
↓DFPCDLHQF ↑TTKEKVLIL ↑LMEKYPVTF LYSFLTPHR LTREPYHTI
↓↑DPKDFPSDV ↓TTKEKAQIV ↑VMEKYSVTF AGHNLYSFL ↓LTRNPYYII
↓DPKDFPCDL ↓TTKEKAQML KLMEKYPVT CAGHNLYSF ↓LTKEPYHTI
REVEDPKDF ↓TTKEKALIL KVMEKYSVT MCAGHNLYS ↓LTKDPYHTI
KKKREVEDP ↑LYKKVMEKY VCAGHNIIF YSALTRNPY
PKKKREVED ↑HAVFSNRTL ↑VYKKLMEKY ICAGHNIIF YSALTREPY
PPKKKREVE LSHAVFSNR ILYKKVMEK RHVCAGHNI YSALTKEPY
↑TPPKKKREV ↑HQFLSHAVF IVYKKLMEK RHICAGHNI CEKLCTFSF YSALTKDPY
STPPKKKRE ↑HHFLSQAVF MLYKKLMEK RQMCAGHNI NFCEKLCTF YLLYSALTK
124 HSTPPKKKRKVEDPKDFPSDLHQFLSQAVFSNRTLACFAVYTTKEKAQILYKKLMEKYSVTFISRHMCAGHNIIFFLTPHRHRVSAINNFCQKLCTFSFLICKGVNKEYLLYSALTRDPYHTIEESIQ 251

↓MYLEFQYNA ↓MLTNRFNHI
LSMYLEFQY MLTERFNYI
LLLSMYLEF ↓MLTGSFDHI
DVFLLLSMY MLTDRFNHI
DVLLLLGMY REEMLTNRF
KCEDVLLLL REEMLTGSF HILDRMDLI
TKCEDVLLL REEMLTDRF↑YILDKMDLI
252 GGLKEHDFNPEEPEETKQVSWKLITEYAVETKCEDVFLLLGMYLEFQYNVEECKKCQKKDQPYHFKYHEKHFANATIFAESKNQKSICQQAVDTVLAKKRVDTLHMTREEMLTERFNHILDKMDLIF 378

FLHCVVFNV
FDFLHCVVF
↓VIFDFLHCV
↓VRFDFLHCI RGKALNVNL
MDSVRFDFL ↑VPKRRYWLL LDLCRGKAL
↓KMDSVRFDF VVFNVPKRR GLLDLCRGK
379 GAHGNAVLEQYMAGVAWLHCLLPKMDSVIFDFLHCIVFNVPKRRYWLFKGPIDSGKTTLAAGLLDLCGGKALNVNLPMERLTFELGVAIDQYMVVFEDVKGTGAESKDLPSGHGINNLDSLRDYL 503

KTLHARFVR
↑VPKTLHARF
↓YPVPKTLHA
↓YPGPKTLQA
↓YAVPKTLQA LLLIWSRPV
NEYPGPKTL LLLLLIWSR
NEYAVPKTL TLLLLLIWS
NKYPVPKTL ITLLLLLIW
↓NDYPVPKTL ↓GITLLLLLI
TMNEYPGPK SGITLLLLL
TMNEYAVPK ↓IHTYFRPKI LQSGITLLL
TMNKYPVPK ↑ARFLIHTYF ILQSGITLL
TMNDYPVPK LHARFLIHT ↓RILQSGITL IWSRPVADF
504 DGSVKVNLEKKHLNKRTQIFPPGLVTMNEYPVPKTLQARFVRQIDFRPKIYLRKSLQNSEFLLEKRILQSGMTLLLLLIWFRPVADFATDIQSRIVEWKERLDSEISMYTFSRMKYNICMGKCIL 628

629 DITREEDSETEDSGHGSSTESQSQCSSQVSDTSAPAEDSQRLDPHSQELHLCKGFQCFKRPKTPPPK*

Frequency of 9mer variants (%)			
<1%	1% – <5%	5% – <10%	10% – < 25.0%
